# Brain-Specific SNAP-25 Deletion Leads to Elevated Extracellular Glutamate Level and Schizophrenia-Like Behavior in Mice

**DOI:** 10.1155/2017/4526417

**Published:** 2017-11-28

**Authors:** Hua Yang, Mengjie Zhang, Jiahao Shi, Yunhe Zhou, Zhipeng Wan, Yicheng Wang, Yinghan Wan, Jun Li, Zhugang Wang, Jian Fei

**Affiliations:** ^1^School of Life Science and Technology, Tongji University, Shanghai 200092, China; ^2^Shanghai Engineering Research Center of Model Organisms (SRCMO/SMOC), Shanghai 201203, China

## Abstract

Several studies have associated reduced expression of synaptosomal-associated protein of 25 kDa (SNAP-25) with schizophrenia, yet little is known about its role in the illness. In this paper, a forebrain glutamatergic neuron-specific SNAP-25 knockout mouse model was constructed and studied to explore the possible pathogenetic role of SNAP-25 in schizophrenia. We showed that SNAP-25 conditional knockout (cKO) mice exhibited typical schizophrenia-like phenotype. A significantly elevated extracellular glutamate level was detected in the cerebral cortex of the mouse model. Compared with Ctrls, SNAP-25 was dramatically reduced by about 60% both in cytoplasm and in membrane fractions of cerebral cortex of cKOs, while the other two core members of SNARE complex: Syntaxin-1 (increased ~80%) and Vamp2 (increased ~96%) were significantly increased in cell membrane part. Riluzole, a glutamate release inhibitor, significantly attenuated the locomotor hyperactivity deficits in cKO mice. Our findings provide *in vivo* functional evidence showing a critical role of SNAP-25 dysfunction on synaptic transmission, which contributes to the developmental of schizophrenia. It is suggested that a SNAP-25 cKO mouse, a valuable model for schizophrenia, could address questions regarding presynaptic alterations that contribute to the etiopathophysiology of SZ and help to consummate the pre- and postsynaptic glutamatergic pathogenesis of the illness.

## 1. Introduction

Schizophrenia (SZ), a complicated psychiatric disorder, affects almost 1 percent of the general population in the world [1, 2]. While the etiology and pathophysiology of SZ remain elusive, genetic risk factors are recognized as an important contributing factor to the pathogenesis of this neuropsychiatric disorder [3]. It has been documented that the synaptosomal-associated protein of 25 kDa (SNAP-25) is a candidate risk gene for SZ, as supported by the following lines of evidence: (1) Genetic association and linkage studies have revealed that chromosome region 20p12.2 which SNAP-25 locates in has significant linkage with SZ [4, 5]. (2) Large-scale genome-associated case-control studies have revealed that several single nucleotide polymorphisms (SNPs) of SNAP-25 are significantly associated with SZ [6]. (3) Various postmortem analyses have found that the expression of SNAP-25 is reduced in prefrontal cortex (PFC) and hippocampus in brain of patients with SZ [7–9]. However, how the reduction of SNAP-25 level is involved in the pathological phenotype remains unknown.

In the brain, SNAP-25 proteins are abundantly expressed in glutamatergic terminals, while relatively lower amounts of the protein are detectable in GABAergic terminals [10]. The primary role of SNAP-25 is a fundamental component of soluble N-ethylmaleimide-sensitive factor attachment protein receptor (SNARE). Together with cell membrane protein syntaxin and vesicle-associated membrane protein (VAMP), SNAP-25 mediates presynaptic vesicle docking and exocytosis [11]. In addition to regulation of synaptic transmission, SNAP-25 is also believed to regulate intracellular calcium dynamics through negative modulation of voltage-gated calcium channels. It also plays a role in other neuronal processes, including spine morphogenesis, postsynaptic receptor trafficking, and neuronal plasticity [12]. But the underlying cellular mechanisms still need to be explored.

Over the past few decades, several mouse models have been constructed to elucidate the physiological role of SNAP-25 *in vivo*. Complete knockout of SNAP-25 in mice leads to no evoked exocytosis and death of the animal at birth. However, heterozygous mice are able to survive and exhibit locomotor hyperactivity and learning deficiencies. SNAP-25 knock-in mice, which have a single amino acid substitution of Ala for Ser187, have been shown to display epilepsy and anxiety-related behavior. The blind-drunk (Bdr) mouse expresses a dominant point mutant SNAP-25b protein, resulting in impaired sensorimotor gating and ataxia, while SNAP-25b-deficient model has developmental defects, seizures, and impaired synaptic plasticity. Conclusively, SNAP-25 mouse mutants occupied a series of psychophenotypes. However, the results derived from different models were inconsistent or conflicting, and no convincing evidence has supported an association between SNAP-25 and SZ. Therefore, additional investigations are necessary to demonstrate the possible role of SNAP-25 in SZ.

Taking into account that the cerebral cortex and hippocampus are the critical brain areas associated with SZ, brain-specific SNAP-25 knockout mice are most suitable to explore the relationship between SNAP-25 and SZ. Furthermore, SNAP-25 has been highly expressed both in neuron and endocrine cells, so brain-targeted SNAP-25 modification is capable of excluding interference signals from the surrounding system. In this study, we designed a brain-specific SNAP-25 knockout mouse, through behavioral phenotyping, molecular detection, and drug treatment within this model, to explore the possible pathogenetic role of SNAP-25 in SZ.

## 2. Materials and Methods

### 2.1. Animals

Mice were maintained in a specific pathogen-free (SPF) facility under a 12 h light/dark cycles. All animal protocols were approved by the Institutional Animal Care and Use Committee at Shanghai Research Center for Model Organisms (number 2015-0005). Mice were sacrificed with carbon dioxide when experiments were completed.

### 2.2. Generation of SNAP-25 cKO Mice

Genomic DNA of SCR012 ES cells isolated from 129S6/SvEv mouse strain was utilized to amplify SNAP-25 homologous fragments. The targeting strategy is flanking exon 4 of the mouse SNAP-25 gene with two loxP cassettes. Chimera mice were constructed by injecting recombination-positive ES cells into blastocyst and backcrossed to C57BL/6J mice. SNAP-25^L2/L2^: CaMKII*α*-Cre^+/wt^ mice were obtained by regular breeding procedure between SNAP-25^L2/L2^ mice and CaMKII*α*-Cre strain (The Jackson Laboratory, stock number 005359) in which Cre-recombinase is extensively expressed in forebrain excitatory neurons by p20 [13, 14]. PCR genotyping was performed with genomic DNA isolated from mouse tail tissue. The primers used for genotyping were designed for identifying loxP (forward: 5′-CACTGCAGAGATTGCAGTATCACTA-3′, reverse: 5′- CAATGCACAGTTATTGTATTGAAGG-3′), and Cre sequences (forward: 5′- AGCGATGGATTTCCGTCTCTGG-3′, reverse: 5′- AGCTTGCATGATCTCCGGTATTGAA -3′).

### 2.3. Western Blot Analysis

Cell cytosolic or membrane protein lysates of mouse brain tissues were prepared using Mem-PER Plus Membrane Protein Extraction Kit (Thermo Scientific, 89842). Then, the lysates were separated by SDS–PAGE and probed with specific antibodies: SNAP-25 (Abcam, ab66066), SNAP-23 (Abcam, ab3340), syntaxin (Santa Cruz, sc-12736), Vamp2 (Abcam, ab6276), Munc-18 (SYSY, 116002), Phospho-Synaptotagmin (R&D Systems, PPS085), *β*-ACTIN (Abcam, ab6276), TUBULIN (Abcam, ab15246), and Na/K ATPase (Millipore, 05-369). For quantification, the densitometry measurement of each band was first normalized to that of *β*-ACTIN, TUBULIN, or Na/K ATPase (used as loading control) and then averaged from at least three independent samples.

### 2.4. Immunofluorescence Staining

Sagittal brain sections (15 *μ*m in thickness) were prepared from a fixed brain with 4% paraformaldehyde, and immunostaining was performed as described [15]. Antibodies used for the immunostaining were SNAP-25 (Abcam, ab66066) and VGLUT1 (SYSY, 135304). Fluorescence was analyzed on a Nikon A1R confocal microscope (Nikon Instruments, Shanghai, CN).

### 2.5. Behavioral Testing

Behavioral phenotyping was performed on age-paired adult male mice (8 to 12 weeks for both cKO and Ctrl littermates). Prior to testing, mice were habituated to the testing room for 2 h.

#### 2.5.1. Open-Field Test

The open field is a square arena (40 × 42 × 30 cm). 8 cm width elongated area along the wall is defined as the “peripheral zone,” approximately 66% of the total area. We place the mouse in the center of the box and enable it to move freely for 15 mins, and an infrared tracking system (Kinder Scientific, Julian, USA) was borrowed to record the movement.

#### 2.5.2. Prepulse Inhibition of the Startle Response

The Acoustic Startle Reflex Starter Package and Startle Reflex 5 software system (Med Associates Inc., St. Albans, VT) was used to assess prepulse inhibition (PPI). The test began with placing the mouse in the chamber's cylinder to acclimate for 5 mins. The remainder of the test consisted of two blocks of trials. A 65 dB background sound was presented throughout the session. The first block consisted of twenty trials of 20 ms and 105 dB sound served as startle stimuli and presented with different intertrial intervals (10–30 s). The second block consisted of 50 trials, with five varying trial types: startle only, or a 10 ms prepulse sound at 70, 75, 80, and 85 dB appeared 50 ms before the startle stimulus. The trial types were presented in random order with intertrial interval range from 10 to 30 s. Percent PPI of the startle response was calculated as the following formula: [1 − (startle response to prepulse + startle/startle response to startle only)] × 100.

#### 2.5.3. Social Approach-Avoidance

The test was carried out as previously described [16]. Approach-avoidance behaviors toward an unfamiliar social partner were recorded by an infrared tracking system. The arena was a plastic open field (42 × 42 cm) containing an empty wire mesh cage (10 × 8 cm) located at one side of the field. During the first session (“no target”), the experimental mouse was introduced into the field and its trajectory was tracked for 5 mins. During the second session (“target 1”), the conditions were identical except that a social target animal (an unfamiliar C57BL/6J male mouse) had been introduced into the cage, and for the third session (“target 2”), the social target mouse was an unfamiliar C57BL/6J female. The tracking data from both the “no target” and “target” conditions were used to determine the time spent by the experimental mouse in the “interaction zone” (an 8 cm wide corridor surrounding the cage) and in the “corners” of the open field opposite to the location of the cage.

#### 2.5.4. Hole-Board Test

The apparatus was a white wooden board (25 × 25 cm) with 16 evenly spaced holes. The number of head-dips was recorded by KS motor infrared monitor system over a 30-minute period.

#### 2.5.5. Nest Building Assay

The nest building test was performed as previously described [17]. Briefly, one square piece of material made of cotton fibre (5 × 5 cm) was put in a cage with an individual mouse. Pictures of the nests were taken 16 h later. The quality of the nest was assessed using the following score: 1, nest not noticeably touched; 2, nest partially torn up; 3, mostly shredded but not identifiable nest site; 4, an identifiable but flat nest; 5, a well-defined nest with walls.

#### 2.5.6. Passive Avoidance Task

The apparatus employed in the passive avoidance task is composed of compartment shuttle chambers, one dark with shock generator and other illuminated compartments. In acquisition trials (2 days), the mouse was allowed to explore the apparatus freely for 5 mins. It would encounter an electric shock (0.5 mA, 2 s duration) once it was inside of the dark chamber with all four paws. On the third day of the trial, the mouse was positioned in the illuminated compartment. Its latency to enter the dark compartment (step-through latency) was recorded automatically.

### 2.6. *In Vivo* Brain Microdialysis

*In vivo* brain microdialysis was carried out to measure glutamate content in the extracellular fluid in the cerebral cortex as previously described [18]. After mice had been anesthetized with inhaled isofluorane (3%), the cerebral cortex was surgically exposed and a microdialysis probe (MAB6.14.2) was inserted into the following coordinates relative to the bregma in mm: −2 to the anterior/posterior axis, ±2.0 to the lateral/medial axis, and −2.5 to the dorsal/ventral axis. Microdialysis was performed by perfusing of the probe with artificial cerebrospinal fluid at a flow rate of 2 *μ*L/min via a microinfusing pump. The total volume of each dialysate sample (20 mins) was 40 *μ*L. Samples were stored at −80°C until use.

### 2.7. Preparation of Tissue Samples for HPLC

Cerebral cortex and hippocampus tissues were isolated from the brains of *SNAP-25* cKO mice and their age- and sex-paired control littermates. After weighing, the samples were homogenized in ice-cold 0.4 M HClO_4_ and centrifuged at 10,000*g* for 15 mins at 4°C. Then, 1 *μ*L of supernatant was mixed with 750 *μ*L of 2 M KHCO_3_ and centrifuged at 3,000*g* for 5 mins. Supernatant was gathered and stored at −80°C until use.

### 2.8. High-Performance Liquid Chromatography (HPLC)

HPLC analyses were performed in the State Key Laboratory of Medical Neurobiology of Fudan University as previously described [19]. The Agilent 1260 series neurotransmitter analyzer (Agilent Technologies, Santa Clara, CA) was utilized to detect the concentrations of amino acid neurotransmitters. Peaks and relative concentrations were identified by comparison to known external standards (Sigma-Aldrich).

### 2.9. Drug Treatments

Clozapine and LY354740 were purchased from Sigma-Aldrich (St. Louis, MO, USA), and lamotrigine was the product of Glaxosmithkline (Brentford, Middlesex, UK). For stock solutions, clozapine was dissolved in 0.1 M HCl and buffered with NaOH to achieve a final pH of 6.5–7.5. Riluzole was suspended in 10 *w*/*v*% cyclodextrin/saline, and LY354740 or lamotrigine was dissolved in saline. Vehicle was developed in an identical manner without the addition of drug, respectively. Concentrated aliquots of both drugs and vehicles were stored at −20°C. On the day of dosing, aliquots were thawed and diluted to their final concentration in sterile saline. Vehicles or clozapine (2.5 mg/kg), riluzole (10 mg/kg), and LY354740 (15 mg/kg) were injected intraperitoneally into age-matched male mice (8–12 weeks old), respectively, and submitted to the open-field test 30 mins later. Lamotrigine was administered to mice by gavage at a dose of 60 mg/kg per day for 2 weeks, followed by behavior testing.

The dose of drug was selected according to previously used doses in mouse behavioral studies [20–23] and our preliminary tests.

### 2.10. Statistical Analysis

Results are shown as the mean ± SEM. Student's *t*-test was utilized to compare two means and two-way ANOVA followed by Bonferroni test to compare multiple means. The nest building scores were treated as nonparametric data, and statistical analysis was performed using Kruskal-Wallis one-way analysis on ranks followed by multiple comparison using Dunn's method. All statistical analyses were performed using Excel 2010 (Microsoft) or GraphPad Prism 5.0. *P* < 0.05 was examined statistically significant.

## 3. Results

### 3.1. Generation of SNAP-25 Forebrain-Specific KO Mice

**Figure 1:**
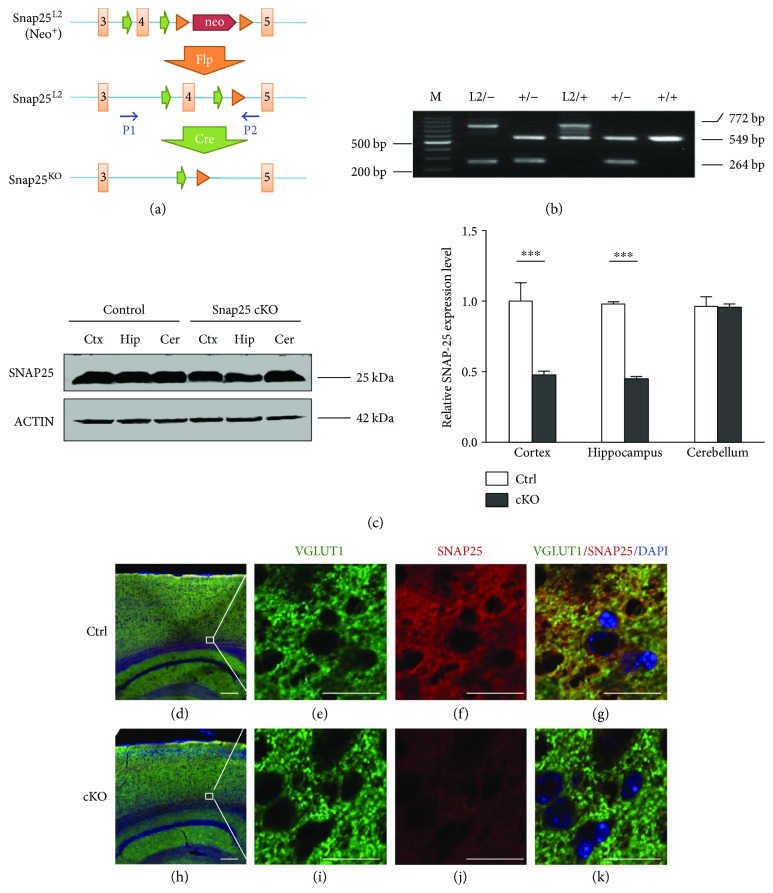
Generation of SNAP-25 forebrain-specific KO mice. (a) Targeting strategy used for the deletion of the SNAP-25 exon 4. P1-2 referred to PCR primers for genotyping, which is located on intron flank exon 4 separately. (b) PCR genotyping of recombinant SNAP-25 locus. (c) Western blot analysis of brain extracts of Ctrl and mutant mice. Right panel: quantitative analysis of western blot images. *n* = 3 per group. ^∗∗∗^*P* < 0.001 compared with control littermates. (d–k) Immunostaining with anti-SNAP-25 (red) and anti-VGLUT1 (green) of cortex of sagittal sections from adult mice brains. Scale bars are 20 *μ*m in (d) and 200 *μ*m in (e).

We generated the SNAP-25-floxed mouse strain SNAP-25^L2/L2^ through inserting of loxP cassettes in the flank sequence of exon4 loci, which caused a frame shifting by Cre-loxP recombinant mechanism (Figure 1(a)). The mouse strain was crossed with CaMKII*α*-Cre transgenic mice to generate forebrain-specific SNAP-25 cKO (SNAP-25^L2/L2^: CaMKII*α*-Cre^+/wt^) model. As we expected, the PCR product of wild-type SNAP-25 allele was 549 bp, whereas the floxed SNAP-25 allele (L2) was 772 bp and the knockout one (L-) was 264 bp. The accuracy of fragments was verified by sequencing (Figure 1(b)). SNAP-25 deletion in different brain areas was confirmed at protein levels. Dramatic reduction of SNAP-25 expression was observed in the cortex and hippocampus but no obvious change in the cerebellum of cKO mice (Figure 1(c)). Furthermore, we executed immunofluorescence examination with anti-SNAP-25 and anti-VGLUT1 (glutamatergic neuron marker) staining. It was found that abundant SNAP-25-positive glutamatergic neurons were detected in the cerebral cortex of Ctrl mice, whereas little staining was found in those of cKOs, confirming that SNAP-25 was inactivated in forebrain glutamatergic neurons (Figures 1(d), 1(e), 1(f), 1(g), 1(h), 1(i), 1(j), and 1(k)).

### 3.2. SNAP-25 cKO Mice Exhibit SZ-Like Phenotype

**Figure 2:**
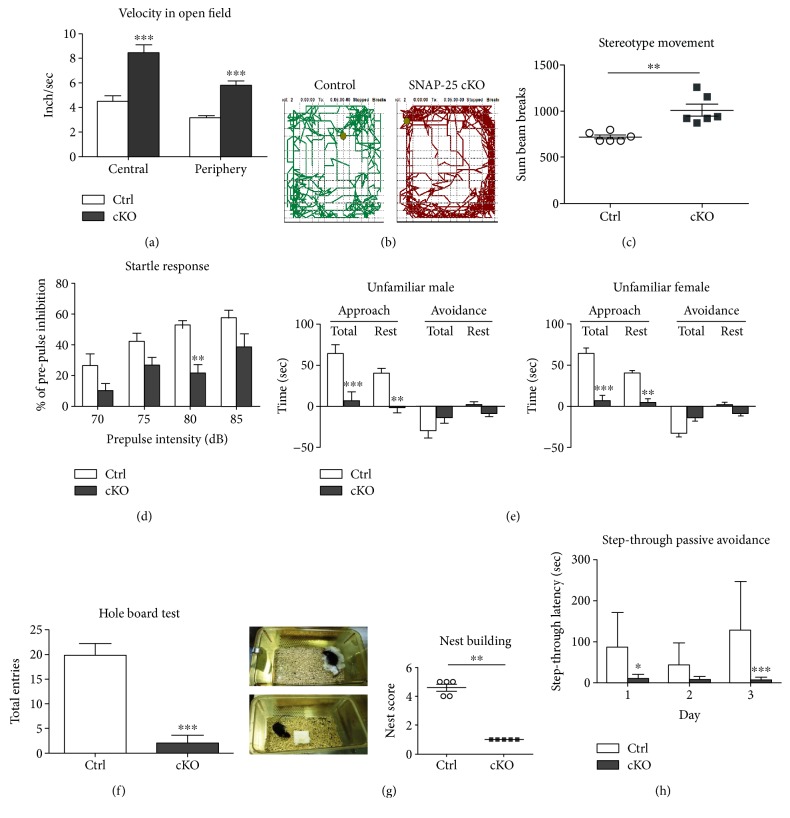
SNAP-25 cKO mice exhibit schizophrenia-like phenotype. (a–d) Summary plots of velocity, movement trajectory, stereotype movement in the open-field test (*n* = 6), and reduced prepulse inhibition (*n* = 5). (e) cKOs display deficient social skills as shown by social approach-avoidance test (*n* = 10). (f) cKOs occupy an impaired tendency to explore novel environment in hole-board test (*n* = 6). (g) Nest building. Left panel: pictures show the results of nesting of different genotypic mice. Right panel: statistical results of nesting scores (*n* = 5). (h) cKOs display impaired learning and memory in the step-through passive avoidance task (*n* = 10). ^∗^*P* < 0.05, ^∗∗^*P* < 0.01, and ^∗∗∗^*P* < 0.001 compared with control littermates.

To determine whether SNAP-25 cKO mice occupy behavioral impairments, we subjected these animals to a battery of behavioral tests. First, in the open-field test, cKO mice showed a significant increased locomotion (3.326 ± 0.160 versus 5.879 ± 0.334 inch/sec, *P* < 0.0001, *n* = 6) and remarkably enhanced stereotype movements (718.50 ± 20.74 versus 1012.00 ± 64.42 breaks, *P* < 0.05, *n* = 6) compared with their control littermates (SNAP-25^L2/L2^, Ctrl, hereafter), demonstrating the abnormal hyperactivity and stereotypical behavior of cKOs (Figures 2(a), 2(b), and 2(c)). Acoustic startle test revealed that prepulse inhibition (PPI) was significantly decreased in the group of cKOs compared to Ctrls [F (1, 8) = 24.37, *P* < 0.0001, *n* = 5], and Bonferroni's post hoc comparison showed a significant disruption of PPI at the prepulse level of 80 dB (*P* < 0.01) (Figure 2(d)). There was no significant difference in the startle response between cKOs and Ctrls, suggesting no apparent hearing deficit (data not shown). Thus, hyperactivity, enhanced stereotypical movements and reduced PPI of SNAP-25 cKO mice fit into the positive symptoms of SZ.

For social behavior judgement, first, we used the social approach-avoidance test to probe animals for their voluntary initiation of social interaction. When presented with an unfamiliar partner, Ctrls had the tendency to spend more time interacting socially, but cKOs displayed intense aversive responses and spent less time in close proximity to the stranger (Figure 2(e)). Also, the significantly reduced head dipping times of cKOs in the hole-board test (19.83 ± 2.39 versus 1.67 ± 1.31 entries, *P* < 0.0001, *n* = 6) also reflected an impaired tendency to explore a novel environment (Figure 2(f)). By mating two genotypic females with wild-type C57BL/6J males, paired for a 4-month period, we observed that both the pregnancy rate and survival pups of cKO females were significantly lower than Ctrls, but the litter size per pregnant mother showed no difference between two groups (6.33 ± 0.47 versus 6.25 ± 0.63 pups/mother, *P* = 0.92, Ctrls *n* = 9, and cKOs *n* = 4), which indicates that both mating and maternal nursing behaviors were defective in cKOs (Table 1). While Ctrls could build clean and typical nests after 16 h with the nesting material, the nests of cKO mice were poorly formed. Substantially decreased nesting score of cKO mice demonstrated their impaired self-care ability (Figure 2(g)). Collectively, the results of the above behavioral tests showed that impaired social skills, exploratory tendency, self-care, and nursing abilities have occurred in cKOs. The cKO mice thus fit the criteria established for negative symptoms of SZ.

To determine whether cKOs have deficits in hippocampus-dependent learning and memory processes, we subjected mice to the step-through passive avoidance task. During the 3-day experiment, cKOs stepped faster into the darker-shock chamber than Ctrls. After subjected to electric shock, the step-through latencies of cKOs were more statistically pronounced compared with Ctrls, which indicated impaired learning and memory of cKOs (Figure 2(h)).

### 3.3. Elevated Glutamate Level in the Cortex of SNAP-25 cKO Mice

**Figure 3:**
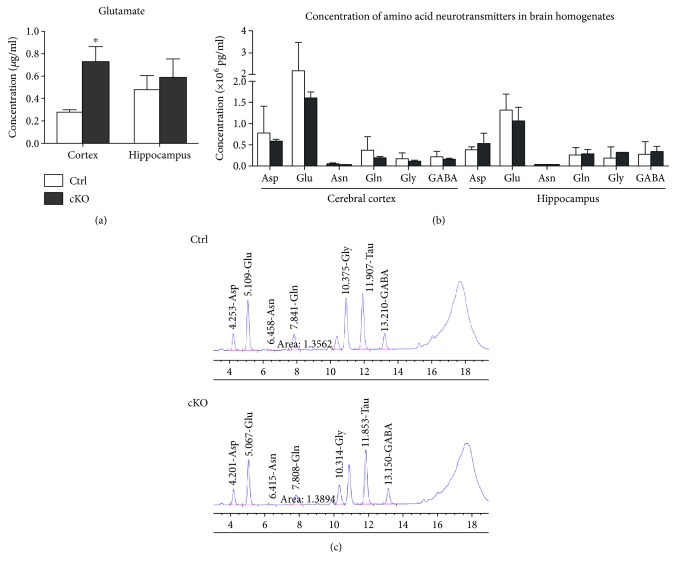
Elevated cortex glutamate level of SNAP-25 cKO mice. (a) The concentration of glutamate (*μ*g/mL) in the microdialysis fluid (Ctrls, *n* = 5; cKOs, *n* = 6). (b) The concentration of amino acid neurotransmitters (×10^6^ pg/mL) in brain homogenates (*n* = 5 per group). (c) The original representative HPLC figures of different genotypic mice. ^∗^*P* < 0.05 compared with control littermates. Asp: aspartate; Glu: glutamate; Asn: asparaginate; Gln: glutamine; Gly: glycine; GABA: *γ*-aminobutyric acid.

We measured the content of glutamate in cerebral cortex and hippocampus of two mice groups by combining *in vivo* microdialysis and HPLC. As indicated in Figure 3(a), a significant increase in glutamate concentration was detected in the microdialysis fluid of the cerebral cortex (0.27 ± 0.02 versus 0.72 ± 0.14 *μ*g/mL, *P* < 0.05, Ctrls *n* = 5, and cKOs *n* = 6), while unchanged level were inspected in hippocampus area (0.48 ± 0.12 versus 0.58 ± 0.17 *μ*g/mL, *P* = 0.63, Ctrls *n* = 5, and cKOs *n* = 6) in cKOs compared with Ctrl mice. However, there was no observable alteration between the concentration of amino acid neurotransmitters of homogenates freshly prepared from the same brain subregions of the cKO and Ctrl mice (Figure 3(b)).

**Figure 4:**
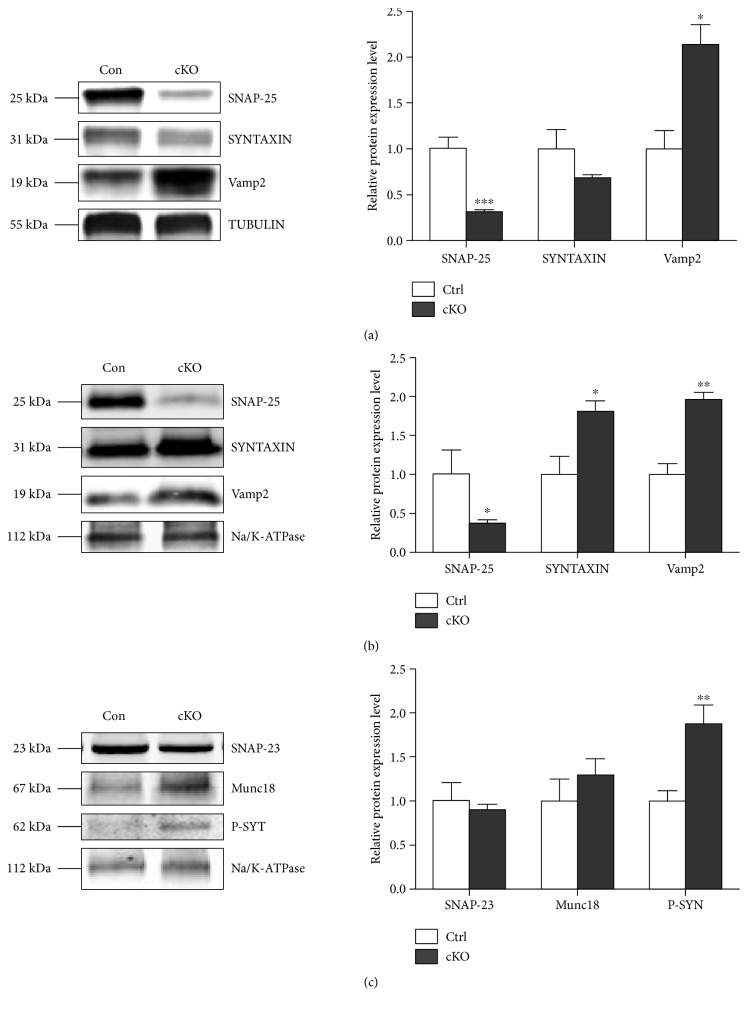
Alteration of expression pattern of SNARE-related proteins. Representative western blot (left) and densitometric analysis (right) of proteins in the cytosolic (a) and membrane (b and c) fractions prepared from mouse cerebral cortex (*n* = 3 per group). ^∗^*P* < 0.05, ^∗∗^*P* < 0.01, and ^∗∗∗^*P* < 0.001 compared with control littermates. P-SYT: phosphorylated synaptotagmin-1.

Subsequently, by using TUBULIN and Na/K ATPase as loading controls, respectively, we examined the expression level of all three SNARE members in the cytoplasm and cell membrane fraction of the cerebral cortex. Compared with Ctrls, SNAP-25 was dramatically reduced around 60% both in cytoplasm and membrane fractions of cKOs, while the other two core members of SNARE complex: Syntaxin-1 (increased ~80%) and Vamp2 (increased ~96%) were significantly increased in cell membrane part (Figure 4). There was no difference in expression of SNAP-25 homologous molecule—SNAP-23 or another important SNARE member—Munc-18 in the cell membrane of the cerebral cortex between cKOs and Ctrls. However, the expression of presynaptic calcium sensor protein—phosphorylated synaptotagmin-1—was significantly elevated about 93% in the cell membrane of the cerebral cortex of cKOs.

### 3.4. Antipsychotic Drugs Attenuated Locomotor Hyperactivity Deficits in cKO Mice

**Figure 5:**
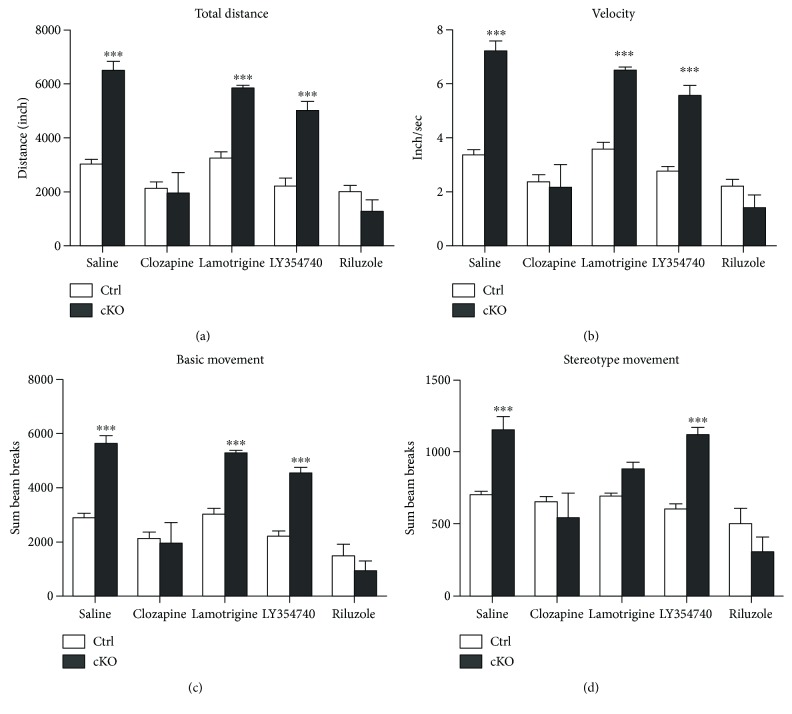
Antipsychotic drugs could attenuate the locomotor hyperactivity deficits in cKO mice. Summary plots of total distance (a), activity velocity (b), basic (c), and stereotype (d) movement in the open-field test. ^∗∗∗^*P* < 0.001 compared with control littermates. Ctrls: *n* = 5; cKOs: *n* = 8.

Antipsychotic drugs, clozapine (atypical schizophrenic drug), lamotrigine (broad-spectrum antiepileptic drug), LY354740 (metabotropic glutamate 2/3 receptor agonist), and riluzole (glutamate release inhibitor) were selected to examine their effects on the locomotor hyperactivities and stereotype behavior of cKO mice. Compared with controls, LY354740 treatment has no detectable effects on all four test index, while lamotrigine could reduce the stereotype movement of cKOs. Administration of either clozapine or riluzole was able to significantly attenuate the hyperactivity and stereotype movements of the SNAP-25 cKO mice (Figure 5).

## 4. Discussion

SNAP-25 is a key molecule involved in synaptic vesicle docking and neurotransmitter release. In line with its central role in neuronal function, it is thought that SNAP-25 is related to human neurological syndromes, especially SZ. In this study, we specifically deleted SNAP-25 gene in forebrain glutamatergic neurons with utilization of the Cre/LoxP strategy. The phenotypes observed in this model fit into SZ-like behaviors, which include positive symptoms (such as hyperlocomotion and reduced PPI), negative symptoms (decreased motivation and impaired social skills), and memory deficit. Our results provided *in vivo* functional evidence to support that altered SNAP-25 expression in the forebrain glutamatergic neurons lead to a greater effect of the illness, confirming the strong association between SNAP-25 and SZ.

It is well known that SNAP-25 plays a key role in medicating neurotransmitter release. Previous studies provided evidence that botulinum neurotoxin type A (BoNT/A) could block synaptic vesicle neuroexocytosis by proteolytic cleavage of SNAP-25, indicating that SNAP-25-deficiency could inhibit neurotransmitter release [24]. However, by *in vivo* brain microdialysis, we found the remarkable elevation of extracellular glutamate levels in cerebral cortex of SNAP-25 cKO mice. No noticeable difference in the total content of amino acid neurotransmitters in the same brain subregions was found between the two groups. Subsequent western blot test revealed the elevated gathering of the SNARE proteins on the cell membrane, which indicated the possibility of increased synaptic vesicle assembly and release. SNAP-25 inactivation seemed not only to fail to block synaptic transmission but also to enhance glutamatergic neurotransmitter in the cortex of cKOs. Previously, Antonucci et al. reported that reduced SNAP-25 levels lead to enhanced evoked glutamatergic transmission in hippocampal cultures and identified that this consequence was not due to changes in a releasable pool of synaptic vesicles [25]. However, we did not detect a statistical difference in hippocampus microdialysis, and we do not know the exact reason yet. We noticed that there were several differences between our and their works: (1) the developmental state of animals (adult mice and E18 mice embryos); (2) the experimental condition (intact animal under physiological condition and *in vitro* cultured cell model); and (3) the detective method (*in vivo* brain microdialysis and whole-cell patch-clamp recording). All above factors may contribute to the inconsistent results we have made.

Thus, the emerging question is how synaptic exocytosis could be enhanced without SNAP-25. To investigate the intrinsic mechanism of this phenomenon, we detected the expression level of three molecules, which are functionally related to SNAP-25 closely. These were (1) SNAP-23, the closest homolog of SNAP-25, which may be the substitution for SNAP-25 to mediate synaptic vesicle fusion [26]; (2) mammalian uncoordinated-18 (Munc-18), which has been found to have dual binding ability to syntaxin-1 and Vamp2, classified as the fourth crucial member of SNARE-pin assembly and may be another alternative for mediating neurotransmitter release [27, 28]; and (3) as the synaptic vesicle Ca^2+^ sensor, synaptotagmin-1 could be phosphorylated with calcium influx and trigger the synaptic release subsequently. Endogenous SNAP-25 negatively modulates neuronal voltage-gated calcium channels (VGCCs) [11, 29]. Therefore, SNAP-25 deficiency may release VGCC activity from SNAP-25-mediated inhibition, thus resulting in exaggerated calcium influx and triggering exocytic release of glutamate. Accordingly, our molecular detection results showed the phosphorylated synaptotagmin-1 was obviously elevated, which indicated the active of VGCCs and enhanced calcium influx that may lead to greater synaptic release at the presynaptic terminal.

Moreover, we assessed the effects of commonly used antipsychotic drugs on the locomotor hyperactivities and stereotype behavior of cKO mice. The atypical antipsychotic clozapine can bind to receptors of serotonin, dopaminergic, and glutamatergic system. Multitarget actions make clozapine one of the most efficacious antipsychotics. It is therefore considered the “gold standard” for the treatment of SZ [30]. In regard to the cortical hyperglutamatergic state within our model, we chose three drugs that aim to inhibit presynaptic glutamate release through different pathways. These were (1) LY354740, a presynaptic metabotropic glutamate receptor 2/3 (mGlu2/3) receptor agonist, which helps suppress the release of neurotransmitters, including glutamate and GABA [31]; (2) lamotrigine, which acts primarily through inhibition of glutamate release via blockade of voltage-sensitive sodium channels and stabilization of neuronal membrane [32]; and (3) riluzole, which has diverse effects on multiple components of the glutamatergic system, such as inhibition of glutamate release by depression of voltage-gated ion channels (sodium, potassium, and calcium) and inhibition of autoreceptors. Riluzole also affects glial cells by increasing glutamate uptake, trafficks with AMPA receptors, and so on [33, 34]. Subsequent open-field test results showed that (1) clozapine could attenuate heightened locomotor activity of cKO mice. Its validity illustrated that SNAP-25 cKO mice could respond effectively to antipsychotics drug, which is the qualifying standard for an animal disease model; (2) glutamate release inhibitors occupied different efficacies on our mice and administration of riluzole has significantly corrected the hyperactivity of the SNAP-25 cKOs, whereas lamotrigine could only alleviate the stereotype movements of the mouse model, and LY354740 did not alter the activity at all. These results suggested that the hyperglutamatergic phenotype of our model may be associated closely with enhanced calcium influx rather than impaired mGlu2/3 receptor function, which corresponded with our previous observation. Further investigations are still required to provide more evidences to explore the detailed mechanisms of elevated extracellular glutamate tones in SNAP-25 cKO mice.

The involvement of glutamatergic mechanism in SZ has been hypothesized for many years. SZ-relative abnormalities have been well documented in mice with mutations in postsynaptic components of glutamatergic transmission, such as NMDAR [35, 36], glycine transporter [37], and metabotropic glutamate receptor [38]. The hypofunction of postsynaptic NMDAR on inhibitory neurons that leads to disinhibition of glutamate transmission and glutamate excitotoxicity has formed the bedrock of the glutamate hypothesis of SZ. However, influence of presynaptic glutamatergic deficits is less well understood. Being a key component of glutamatergic neurotransmission at presynaptic locus, SNAP-25 deficiency induced typical SZ-like behavior demonstrated the strong association between presynaptic dysfunction and the outbreak of SZ. SNAP-25 cKO mice would be a useful novel tool for investigating presynaptic alterations that contribute to the etiopathophysiology of SZ. This research helps to consummate the pre- and postsynaptic glutamatergic pathogenesis of SZ.

## 5. Conclusion

This study showed that the forebrain glutamatergic neuron-specific SNAP-25 cKO lead to a typical SZ-like phenotype. The deficiency of SNAP-25 may lead to enhanced calcium influx and exaggerated glutamatergic release and may result in the elevated extracellular glutamate level. Riluzole attenuates the locomotor hyperactivity deficits in cKO mice. Our results provided new insight that SNAP-25 dysfunction has direct consequences on synaptic transmission and contributes to developmental of SZ. SNAP-25 cKO mouse could be a valuable new model for SZ and could be used to address questions regarding pathophysiology and etiology of the illness.

## Figures and Tables

**Table 1:** Mating and nursing test of SNAP-25 cKO females (crossed with C57BL/6 J male).

Genotype	Total pairs	Pregnant females	Born pups	Mean ± SEM	Survival pups	Mean ± SEM
Ctrl	10	9	57	5.70 ± 0.76	57	5.70 ± 0.76
cKO	10	4	25	2.50 ± 1.05^∗^	13	1.30 ± 0.87^∗∗^

^∗^ *P* < 0.05 and ^∗∗^*P* < 0.01 compared with Ctrl.
